# In vitro evaluation of novel N-acetylalaninate prodrugs that selectively induce apoptosis in prostate cancer cells

**DOI:** 10.1186/1471-2407-14-675

**Published:** 2014-09-18

**Authors:** Christopher A McGoldrick, Yu-Lin Jiang, Marianne Brannon, Koyamangalath Krishnan, William L Stone

**Affiliations:** Department of Pediatrics, East Tennessee State University, Johnson City, TN 37614-0578 USA; Center for Biomedical Imaging, Department of Radiology and Radiological Sciences, Medical University of South Carolina, Charleston, SC 29425 USA; Division of Hematology-Oncology, Department of Internal Medicine, East Tennessee State University, Johnson City, TN 37614 USA

**Keywords:** Prostate cancer, Prodrugs, Chemotherapy, Glutathione, Oxidative stress, Apoptosis, Cell viability, Oxidized protein hydrolase, Reactive oxygen species, Quinone methide

## Abstract

**Background:**

Cancer cell esterases are often overexpressed and can have chiral specificities different from that of the corresponding normal cells and can, therefore, be useful targets for activating chemotherapeutic prodrug esters. Prodrug esters are inactive compounds that can be preferentially activated by esterase enzymes. Moreover, cancer cells often exhibit a high level of intrinsic oxidative stress due to an increased formation of reactive oxygen species (ROS) and a decreased expression of some enzymatic antioxidants. Prodrugs designed to induce additional oxidative stress can selectively induce apoptosis in cancer cells already exhibiting a high level of intrinsic oxidative stress. This study focused on the in vitro evaluation of four novel prodrug esters: the R- and S- chiral esters of 4-[(nitrooxy)methyl]phenyl N-acetylalaninate (R- and S-NPAA) and the R- and S- chiral esters of 4-[(nitrooxy)methyl]naphth-1-yl N-acetylalaninate (R- and S-NQM), which are activated, to varying extents, by oxidized protein hydrolase (OPH, EC 3.4.19.1) yielding a quinone methide (QM) intermediate capable of depleting glutathione (GSH), a key intracellular antioxidant. OPH is a serine esterase/protease that is overexpressed in some human tumors and cancer cell lines.

**Methods:**

To evaluate the chiral ester prodrugs, we monitored cellular GSH depletion, cellular protein carbonyl levels (an oxidative stress biomarker) and cell viability in tumorigenic and nontumorigenic prostate cancer cell lines.

**Results:**

We found that the prodrugs were activated by OPH and subsequently depleted GSH. The S-chiral ester of NPAA (S-NPAA) was two-fold more effective than the R-chiral ester (R-NPAA) in depleting GSH, increasing oxidative stress, inducing apoptosis, and decreasing cell viability in tumorigenic prostate LNCaP cells but had little effect on non-tumorigenic RWPE-1 cells. In addition, we found that that S-NPAA induced apoptosis and decreased cell viability in tumorigenic DU145 and PC3 prostate cell lines. Similar results were found in a COS-7 model that overexpressed active human OPH (COS-7-OPH).

**Conclusions:**

Our results suggest that prostate tumors overexpressing OPH and/or exhibiting a high level of intrinsic oxidative stress may be susceptible to QM generating prodrug esters that are targeted to OPH with little effect on non-tumorigenic prostate cells.

## Background

Numerous observations have shown that cancer cells exhibit a high level of intrinsic oxidative stress due to the generation of high levels of reactive oxygen species (ROS) and the suppression of some antioxidant enzymes [1–4]. The increased ROS generation in cancer cells is not just a metabolic happenstance but is required for many aggressive cancer phenotypes including a disruption of various cell-signaling cascades allowing cells to escape apoptosis [1–3, 5, 6]. Most chemotherapeutic agents kill cancer cells by causing the production of even higher levels of ROS thereby causing oxidative stress induced apoptosis [7].

The increased basal level of oxidative stress in cancer cells is attributable to the activation of the Akt kinase signaling cascade, which increases cellular ROS and impairs of some enzymatic ROS detoxifying mechanisms, as well an increased generation of ROS from NADPH oxidase (Nox) [1, 3]. Akt is a serine/threonine kinase that plays a pivotal role in a diverse set of signaling cascades involved in the regulation of cell survival, cell growth, glucose metabolism, cell motility and angiogenesis [8]. Akt is activated when phosphorylated and activated-Akt normally promotes cell survival by inactivating the components of apoptotic stimuli. However, under oxidative stress conditions the pro-survival function of Akt can be overridden and function in a pro-apoptotic role [9]. Chemotherapeutic agents that induce oxidative stress and produce heightened cellular levels of ROS therefore have the potential to selectively induce apoptosis in Akt-activated cancer cells.

Tumor cell apoptosis can be induced through oxidative stress by reducing or inhibiting cellular antioxidants [7]. Glutathione (L-γ-glutamyl-L-cysteinylglycine or GSH) is the primary intracellular antioxidant and plays a key role in modulating tumor cell proliferation as well as the resistance of tumors to many chemotherapeutic drugs [10]. GSH depletion causes growth inhibition in many types of cancers including pancreatic cancer [11–13]. In an animal model, GSH depletion was found to sensitize melanoma cancer cells to combination chemotherapy and eliminate metastatic disease [11].

**Figure 1:**
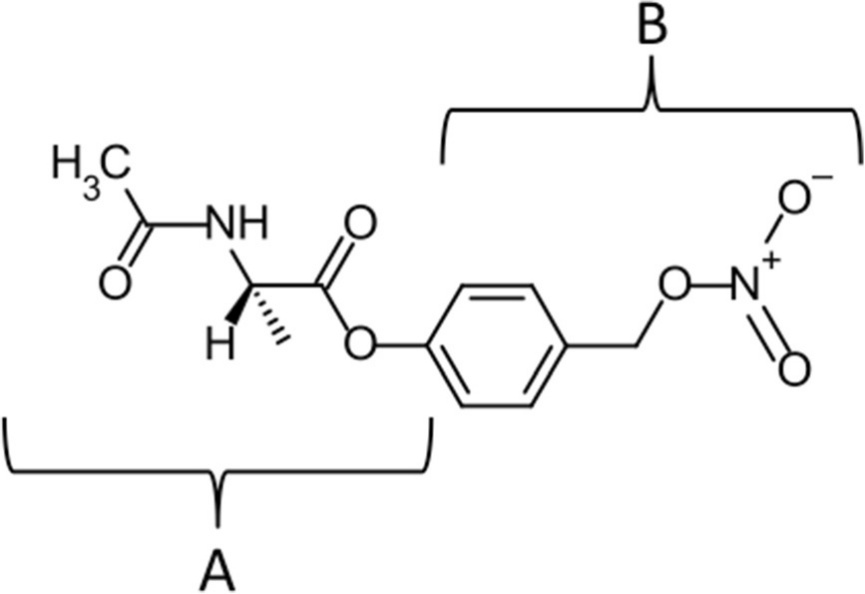
**Moieties of the N-acetylalaninate prodrug.** The N-acetylalaninate prodrugs are hybrids of two esters. We previously demonstrated that the N-acetylalaninate moiety **(A)** is specifically hydrolyzed by OPH in prostate cell lines [24]. The GSH depleting ability of the quinone methide (QM) generating moiety **(B)** is well documented. Combining these two moieties creates an ester substrate that is specifically activated by OPH to generate a QM, which depletes GSH (see Figure 2).

**Figure 2:**
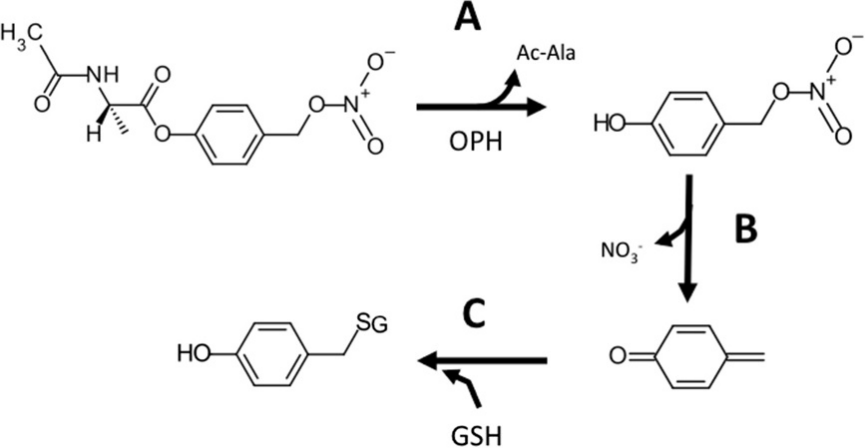
**Mechanism of N-acetylalaninate prodrug activation by OPH and subsequent depletion of glutathione. A)** The ester bond of the prodrug is cleaved by the esterase activity of oxidized protein hydrolase (OPH) releasing acetylalaninate (Ac-Ala) and a (4-hydroxyphenyl)methyl nitrate intermediate. **B)** The intermediate quickly undergoes elimination releasing NO_3_^-^ and forming a reactive quinone methide (QM). **C)** The QM rapidly reacts with the thiol group of reduced glutathione (GSH) in a Michael addition leaving GSH unavailable to participate in cellular redox reactions.

Nitric oxide (NO) donating acetylsalicylic acid (NO-ASA) is a promising anticancer prodrug ester that depletes GSH and promotes oxidative stress induced apoptosis [14–21]. NO-ASA is thought to exert its anticancer effects by an esterase catalyzed release of an electrophile quinone methide (QM) intermediate that selectively reacts with and depletes intracellular GSH [15, 22]. We have hypothesized that a hybrid ester prodrug (see Figure 1) containing the QM generating moiety that is selectively hydrolyzed and activated by oxidized protein hydrolase (OPH) will deplete intracellular GSH (see Figure 2) and promote oxidative stress induced apoptosis in cancer cells by a mechanism similar to that of NO-ASA, i.e., release of a QM depleting intermediate [23].

**Figure 3:**
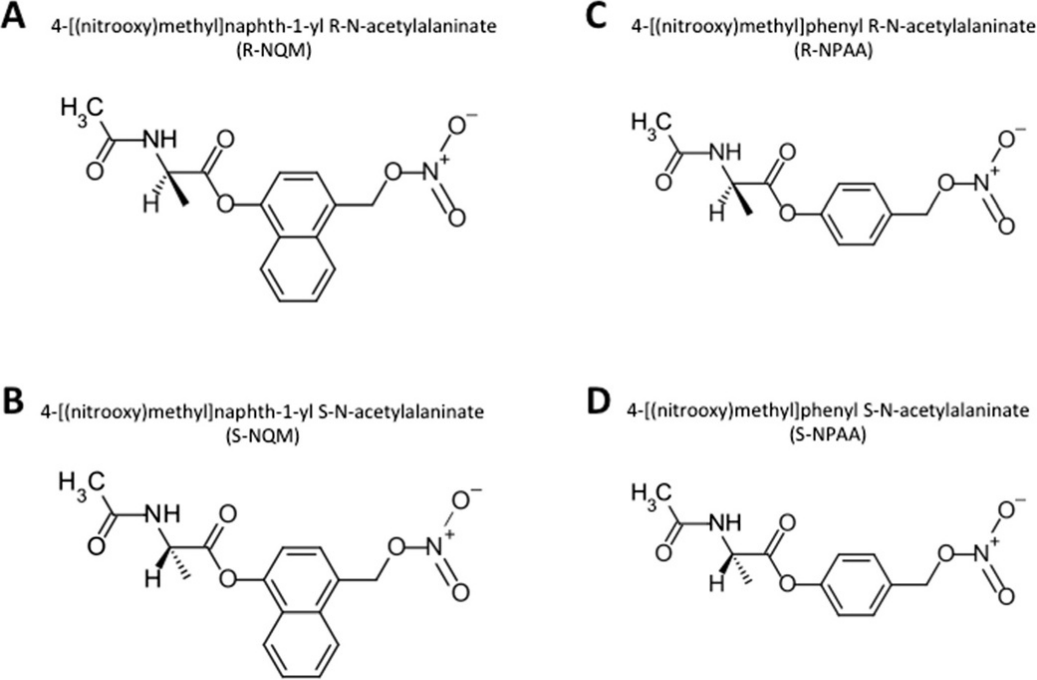
**Structures of chiral N-acetylalaninate prodrugs. A)** R-NQM and **B)** S-NQM are chiral esters designed after α-naphthyl N-acetylalaninate (a known OPH substrate) with the addition of a NO-donating, QM generating moiety. **C)** R-NPAA and **D)** S-NPAA are structurally identical to R-NQM and S-NQM with the exception of a phenyl replacing the naphthyl core of the prodrug.

OPH (EC 3.4.19.1), also called acylamino acid releasing enzyme (or AARE), is a serine esterase/protease that we found to be over expressed in some tumorigenic prostate cell lines [24]. Moreover, histological data in the Human Protein Atlas shows that OPH can be strongly expressed in cases of colorectal, breast, prostate, ovarian, endometrial and liver cancers [25]. We have previously found that OPH selectively catalyzes the hydrolysis of chiral α-naphthyl-N-acetylalaninate (ANAA) esters with a preference for the S-isomer (S-ANAA) [24]. A novel prodrug S-NPAA (Figure 1), was previously advanced as a plausible anticancer prodrug candidate based on its *in silico* binding affinity to the active site of 3-dimensional models of both rat (rOPH) and human OPH (hOPH) as well as its in vitro ability to deplete GSH when activated by rat OPH (rOPH) [23]. S-NPAA is composed of an N-acetylalaninate moiety (indicated as "A" in Figure 1) recognized by OPH and the QM generating moiety of NO-ASA (indicated as "B" in Figure 1). In this study, the effectiveness of the S-NPAA, and three other similar prodrugs (Figure 3), was evaluated in tumorigenic (LNCaP, DU145, PC3) and non-tumorigenic (RWPE-1) prostate cell lines as well as COS-7 cells overexpressing human OPH (COS-7-OPH). We have previously characterized the expression of OPH in LNCaP, RWPE-1, COS-7 and COS-7-OPH cell lines [24]. Moreover, Kumar et al. [3] have characterized the degree of Akt activation in RWPE-1, LNCaP, DU145 and PC3 cells as well as the basal levels of oxidative stress. We found that S-NPAA was the most effective prodrug in its ability to deplete GSH, cause oxidative stress, induce apoptosis, and decrease cell viability, particularly in cell lines overexpressing OPH.

## Methods

### Materials

Reduced glutathione (GSH), digitonin, dimethyl sulfoxide (DMSO), 2,2,2-trichloroacetic acid (TCA), 2,4-dinitrophenylhydrazine (DNPH), 5,5’-dithiobionitrobenzoic acid (DTNB) and diisopropyl fluorophosphate (DFP) were purchased from Sigma Chemical Company (St. Louis, MO). DMEM, KSFM and growth factors, and RPMI 1640 cell medium, penicillin/streptomycin solution, and geneticin (G418) and KB plus DNA ladder, Celltracker blue (7-amino-4-chloromethylcoumarin or CMAC), 10kD spin columns, and EnzChek Caspase-3 assay kit were purchased from Invitrogen (Grand Island, NY). BCA kit and the anti-DYKDDDDK (anti-FLAG) antibody (PA1-984B) were purchased from Pierce (Rockford, IL). Celltiter 96 AQueous One MTS kit, described as the MTS viability assay in experiments, was purchased from Promega (Madison, WI) and contained CellTiter96 Aqueous One Solution composed of a tetrazolium compound [3-(4,5-dimethylthiazol-2-yl)-5-(3-carboxymethoxyphenyl)-2-(4-sulfonyl)-2H-tetrazolium, inner salt (MTS) and an electron coupling reagent (phenazine methosulfate). The Apoptotic DNA ladder kit was purchased from Roche (Indianapolis, IN). All chemicals used for the synthesis of prodrugs were purchased from Sigma-Aldrich (St. Louis, MO), TCI (Portland, OR), Acros Organics (Thermo Fisher Scientific, New Jersey) and Lancaster (Ward Hill, MA) and used without further purification.

### Prodrug synthesis

The N-acetyl-L-alaninate quinone methide precursor, 4-[(nitroxy)methyl]phenyl N-acetyl-L-alaninate (S-NPAA) was synthesized as previously described [23]. R-NPAA, S-NQM, and R-NQM were synthesized with the following modifications. R-enantiomers were synthesized using N-acetyl-D-alanine in place of N-acetyl-L-alanine. The naphthyl core of NQM prodrugs were synthesized by replacing 4-(hydroxymethyl)phenol with 4-(hydroxymethyl)-1-naphthol.

### Cell culture and lysates

Tumorigenic cell lines LNCaP (CRL-1704), DU-145 (HTB-81), and PC-3 (CRL-1435) and the non-tumorigenic cell line RWPE-1 (CRL-11609), and COS-7 cells (CRL-1651) were purchased from American Type Culture Collection (ATCC, Manassas, VA), cultured according to ATCC’s instructions and supplemented with 100 U/ml penicillin and 100 mg/ml streptomycin. Cells were detached from the 75 cm^3^ cell culture flasks after reaching 80% confluence by washing the cells with PBS followed by the addition of 0.25% trypsin. The detached cells were centrifuged at 500 × g for 5 min and washed with PBS to remove trypsin. Cells were centrifuged a second time and pellets stored at -80°C. Cell pellets of each cell line were lysed using 2% (wt/vol) digitonin in PBS on ice with vortexing every two min. After 10 min of incubation on ice, the lysates were centrifuged at 18,000 × g for 5 min at 4°C and the supernatant collected. Protein concentrations were determined with the BCA kit using the manufacturer’s instructions.

### Semi-purified OPH from rat liver

OPH was semi-purified from 100 g of rat liver (rOPH) using the method described by Stone et al. [23]. The pooled semi-purified rOPH was analyzed by mass spectroscopy as described by Stone et al. [23] to verify that no other esterases or proteases were present.

### Overexpression of OPH in COS-7 cells

COS-7 cells were transfected using TransIT-LT1 transfection reagent and the vector pCDNA3.1(+) encoding OPH with a Flag tag using the transfection reagent’s manufacturer’s instructions. COS-7 cells overexpressing OPH (COS-7-OPH) were selected using 1 mg/ml G418 over a three-week period. Cells surviving selection were termed COS-7-OPH for further experiments and were maintained with 1 mg/ml G418.

### Glutathione (GSH) depletion assay

A volume of 180 μl of a freshly prepared solution containing 65 μM GSH and 160 μM of prodrug (or a mixture of 160 μM R-NPAA and 160 μM S-ANAA for inhibition assay) in 50 mM phosphate buffer, pH 6.5 was added to each well of a 96-well plate. Cell lysates containing 90 μg of protein was diluted with 50 mM phosphate buffer, pH 6.5 to a volume of 20 μL (or with 50 μM DFP for inhibition assay). The 20 μL lysate solution was added to each well at the zero min time point of the assay. Immediately after lysate was added, 50 μl of 1.25 mM DTNB was added to the wells for the zero hour time point, and the absorbance at 412 nm was read using a SpectraMax Plus 384 plate reader (Molecular Devices, Sunnyvale, CA). At the other indicated time points, 50 μl of 1.25 mM DTNB was added to the wells, and the absorbance at 412 nm was measured. GSH depletion assays using recombinant human OPH were performed by adsorbing 100 μl anti-FLAG antibody in a 96-well plate overnight at 4°C in carbonate buffer, pH 9.6 at a concentration of 10 μg/ml. The wells were rinsed three times with PBS and blocked for 1 hour with 5% non-fat dry milk in PBS. The wells were rinsed three times and a volume of 100 μl of COS-7-OPH cell lysates containing 120 μg protein was added and incubated at room temperature for 2 hours. The wells were then rinsed five times with PBS and the GSH depletion assay was performed.

### Caspase-3 activity assay

RWPE-1, LNCaP, DU145, PC3, COS-7, and COS-7-OPH cells were grown in 25 cm^3^ cell culture flasks to 80% confluence. The cells were then treated with 25 μM NPAA, 1 μM staurosporine, or DMSO in complete growth medium for 6 hours at 37°C, 5% CO_2_. The growth medium was retained to collect floating cells and the adherent cells were lifted using 0.25% trypsin. Growth medium and cells were combined and centrifuged at 500 × g for 5 min, and the resulting cell pellets were washed with PBS to remove trypsin. The cells were then lysed and the cell lysates tested using the caspase-3 activity assay kit with a 96-well plate according to the manufacturer’s instructions. The fluorescence of the wells was measured using a Flurostar Galaxy Fluorometer (BMG Lab Technologies, Inc., Durham, NC) and expressed as relative fluorescence units per minute (RFU/min).

### Electrospray Ionization-mass spectroscopy (ESI-MS)

A 1.5 ml reaction mixture containing 1.5 ml of 52 μM reduced GSH, 160 μM NPAA, and 1 μg semi-purified rOPH were incubated in 50 mM sodium phosphate buffer at room temperature for 1 hour. A control containing 1.5 ml of 52 μM reduced GSH (GSH control) in 50 mM sodium phosphate buffer was also incubated under the same conditions. The reaction mixture and GSH control were filtered using a 10 kD molecular weight cut-off centrifugal filter to remove the OPH protein. The filtered reaction mixture or GSH control was then added to a 500 μl glass syringe, and infused into the ESI source of the mass spectrometer using the syringe pump at a flow rate of 100 nl/min. The reaction mixture and GSH control were analyzed in positive ion mode by electrospray ionization using a LTQ-XL ion trap mass spectrometer (Thermo Fisher). Real-time screen shots of the chromatograms were captured in the Xcalibur browser, version 3.3. Reduced GSH is known to produce a peak with a m/z = 308 [26]. QM covalently bound to glutathione (QM-GS) has a predicted peak at m/z = 413.4 based on molecular weight calculations in Symyx Draw 3.2 (Softonic, San Francisco, CA).

### DNA ladder assay

LNCaP and RWPE-1 cells were grown in 75 cm^3^ cell culture flasks to 80% confluence and treated with NPAA, staurosporine, or DMSO as previously described. Cells were collected as previously described and were then lysed and processed with the Apoptosis DNA Ladder Kit according to the manufacturer’s instructions. The RWPE-1 and LNCaP sample DNA (3 μg), the staurosporine control (3 μg), and DNA ladder supplied with the kit (1 μg) were mixed with loading buffer and added to a 2% agarose gel containing 1:10,000 dilution of SYBR Safe. The gel was electrophoresed at 75 V for 2 hours in TBE buffer and then photographed under UV light using a ChemiDoc XRS + system with Image Lab software (BioRad, Hercules, CA).

### Protein carbonyl assay

RWPE-1, LNCaP, COS-7, and COS-7-OPH cells were grown in 25 cm^3^ cell culture flasks to 80% confluence. The cells were then treated with NPAA or DMSO and collected as previously described. The cells were then lysed with 2% digitonin in PBS and the protein concentration was determined using the BCA assay kit. For each sample, an aliquot of 50 μl of protein lysate containing 5 μg/μl of protein in PBS was added to two 1.5 ml tubes. One tube was used as the negative control tube. A volume of 200 μl of 10 mM DNPH was added to the sample tube, and a volume of 200 μl of 2.5 M HCl was added to the control tube. The tubes were incubated in the dark at 24°C for one hour. Proteins were precipitated by adding 500 μl of 20% TCA and incubating on ice for 5 min, followed by centrifugation at 10,000 × g for 10 min at 4°C. The supernatant fluid was removed and the protein pellets were suspended in 1 ml of 1:1 (vol/vol) ethanol/ethyl acetate followed by centrifugation at 10,000 × g for 10 min at 4°C. Removal of supernatant fluid, suspension of pellets, and centrifugation were repeated three times. The supernatant fluid was then removed and the protein pellets were dissolved in 300 μl of 6 M guanidine hydrochloride and mixed using a vortex mixer every 10 min for one hour. Aliquots of 200 μl from each tube were added to separate wells of a clear 96-well plate, and the absorbance at 370 nm was measured using a Spectra max plus 384 microplate reader (Molecular Devices, Sunnyvale, CA). The corrected absorbance was calculated by subtracting the absorbance of the well containing the control tube aliquot from the absorbance of the well containing the sample tube aliquot. The amount of protein carbonyls in the sample was calculated using the corrected absorbance for each sample and an extinction coefficient of 2.2 × 10^4^ M^-1^ cm^-1^.

### Cellular GSH depletion

LNCaP, RWPE-1, COS-7, and COS-7-OPH were seeded in triplicate in wells of 96-well cell culture plates at 2 × 10^4^ cells/well. The plate was incubated at 37°C, 5% CO_2_ for 18 hours. The cell medium was removed from each well and 200 μl of cell medium containing 60 μM NPAA was added to each well. The cells were incubated at 37°C in 5% CO_2_ for 30 minutes. The medium was removed and replaced with 100 μL of 10 μM CMAC in PBS for 30 min at 37°C, 5% CO_2_. The staining solution was aspirated, rinsed with PBS, and replaced with 100 μl of PBS. The cells were observed at 100× magnification and digitally photographed using a MOTIC inverted phase contrast microscope equipped with a Nikon Coolpix E4300 4-megapixel camera (Martin Microscope, Easley, SC) using a D350/50X DAPI filter. The percent area threshold of staining was measured using ImageJ, v1.440 (NIH, Bethesda, MD).

### Cell viability assay

The MTS viability assay was used to detect viability of the cells in all experiments. Cells cultured in 96-well plates were treated with cell medium (0.2 ml/well) containing indicated doses of NPAA and incubated at 37°C for the specified amount of time. A volume of 20 μl of CellTiter96 Aqueous One (MTS) solution was then added to each well and plates were incubated at 37°C for 60 min. The absorbance of each well was measured at 490 nm using the SpectraMax Plus 384 plate reader. Viability was expressed as a percentage (%) using the formula: Absorbance of treated cells/ Absorbance of untreated cells × 100.

### Statistics

Data were analyzed by analysis of variance (ANOVA) followed with the Scheffe test for significance with P < 0.05 using SPSS 19.0 for Windows (Chicago, Illinois). Results were expressed as the mean ± SD of at least three experiments.

## Results

### S-NPAA is the most effective N-acetylalaninate prodrug and is activated by OPH

**Figure 4:**
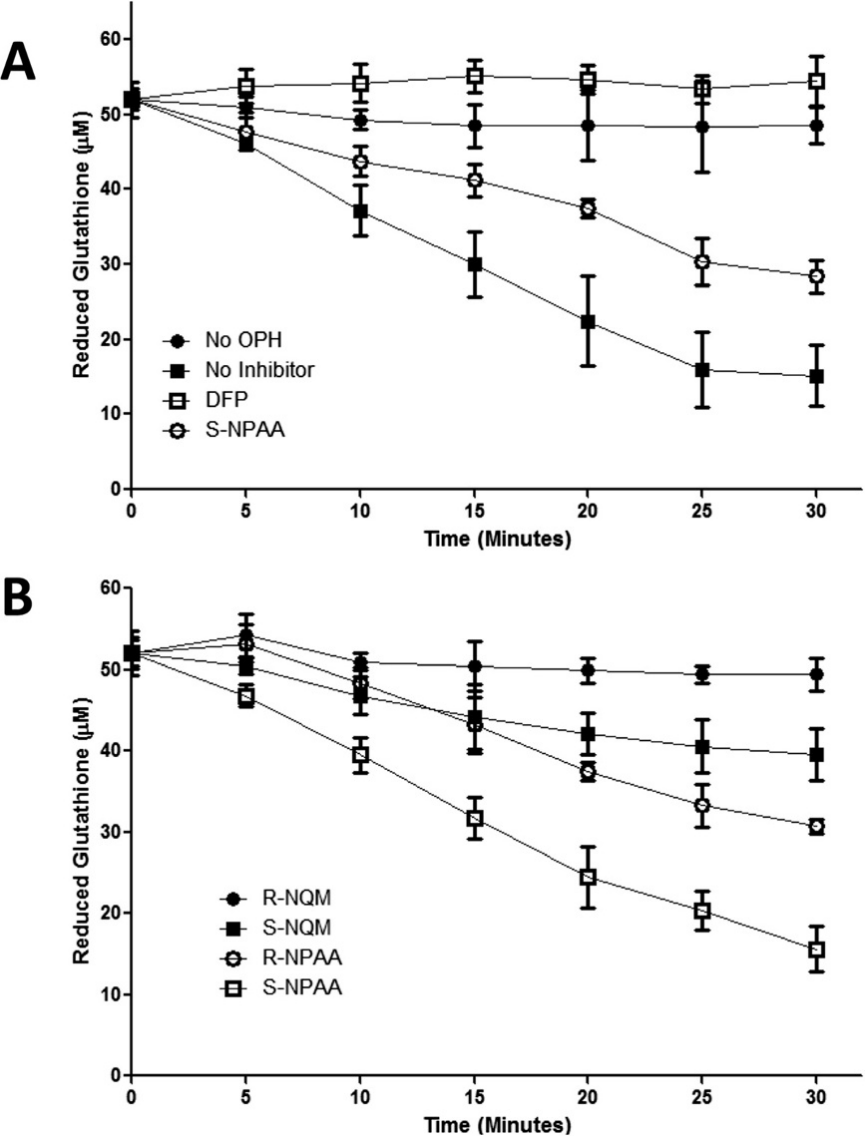
**N-acetylalaninate prodrugs are activated by OPH with a preference for NPAA. A)** A reaction mixture containing reduced GSH, S-NPAA, and the indicated treatment was incubated with or without active human OPH in a 96 well plate. Diisopropyl fluorophosphate (DFP) is an irreversible serine protease inhibitor. At each time point, the amount of reduced GSH was measured as described in the Methods Section. **B)** A reaction mixture containing reduced GSH and the indicated prodrug was incubated with active human OPH in a 96 well plate. At each time point, the amount of reduced GSH was measured as in **A)**. Data points marked with letters that are not the same are significantly different at p < 0.05.

Four chiral N-acetylalaninate ester prodrugs (see Figure 3) were evaluated in this study based on: (1) our previous experimental observations showing that OPH has specificity towards α-naphthyl-N-acetylalaninate substrates [24]; (2) *in silico* protein-ligand binding studies suggesting that S-NPAA has a reasonable affinity to the active site found in predicted three dimensional models of rat and human OPH [23]; (3) structural similarity to NO-ASA which has a toxicology profile superior to that of aspirin [18]. Our first objective was to determine whether the hydrolysis of the newly designed prodrugs was catalyzed, and thus activated, by OPH. We first used an in vitro GSH depletion assay to measure the activation and resulting GSH depletion of the prodrugs by rat liver OPH [23]. As shown in Figure 4A, we found that S-NPAA was hydrolyzed by OPH with an accompanying GSH depletion as anticipated by the mechanism proposed in Figure 2. Moreover, the ability of OPH to activate S-NPAA and deplete GSH was markedly diminished in the presence of the irreversible serine protease inhibitor, DFP, to levels similar to those seen in the absence of OPH. OPH GSH depleting activity was also reduced nearly two-fold when the reaction mixture contained the S-isomer ester of α-naphthyl-N-acetylalaninate (S-ANNA see [24] for structure), an OPH substrate that is not linked to a QM-generating moiety [24]. This result suggests that S-ANAA is acting as a competitive inhibitor of S-NPAA activation by OPH. We found that the prodrugs containing a phenyl moiety (S-NPAA and R-NPAA) were significantly more effective than the prodrugs with a naphthyl moiety (R-NQM and S-NQM) at depleting GSH (see Figure 4B). Because the S chiral ester of NPAA (S-NPAA) was almost two-fold more effective at depleting GSH in vitro than the R-chiral ester (R-NPAA), we chose to focus on S-NPAA for the remaining experiments.

### Activating S-NPAA yields a QM intermediate that covalently reacts with GSH

Hulsman et al. [22] have utilized LC-MS to show that HT29 colon cancer cells incubated with NO-ASA form the expected GSH-QM adduct (see Figure 2). In order to specifically show that GSH depletion by NPAA was similarly caused by the generation of a QM intermediate (as indicated in Figure 2), we used ESI-MS to confirm the presence of the GSH-QM adduct. A reaction mixture was prepared with GSH, S-NPAA, and rOPH in sodium phosphate buffer. The resulting reaction was compared to non-reacted GSH. GSH has a known m/z = 308 [26] and the GS-QM reaction product has a predicted m/z = 413.4. In the control experiment (i.e. no rOPH present) we only found non-reacted GSH with a sharp peak at m/z = 308 while a peak at m/z = 413.4 was observed when rOPH was present indicating the formation of the expected GSH-QM product. The rat liver OPH used in this experiment was semi-purified but still had some minor additional proteins present that were all identified by reverse phase nanospray LC-MS/MS and none were proteases or esterases.

### S-NPAA crosses the plasma membrane and depletes cellular GSH in cells containing high OPH activity

**Figure 5:**
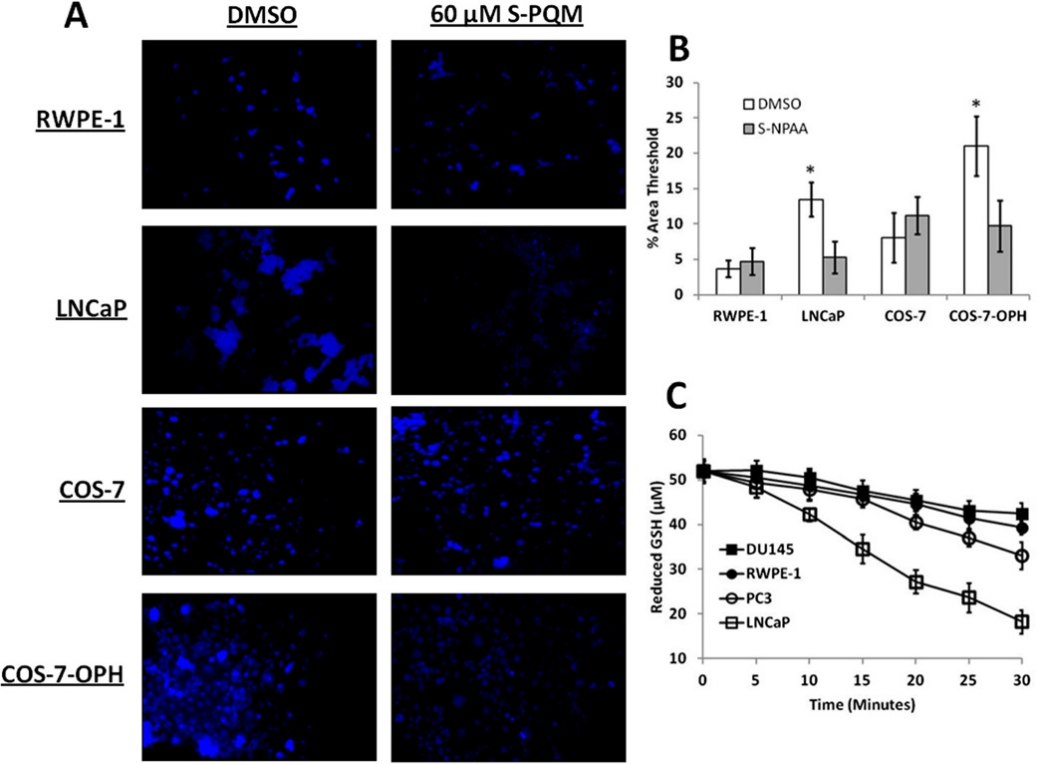
**GSH depletion in cultured cells and prostate cell lysates treated with S-NPAA. A)** LNCaP, RWPE-1, COS-7, and COS-7-OPH cell cultures were incubated with 25 μM S-NPAA for 30 min followed by a 30 min incubation with CMAC. The blue fluorescence indicates the presence of GSH. **B)** Microscopy images were analyzed with ImageJ to measure the relative fluorescence between cell lines. Percent area threshold was defined as the percent area of fluorescence that exceeded background; *indicates that the treatment was significantly different from control (vehicle) at P < 0.05. **C)** A reaction mixture containing reduced GSH, S-NPAA, and 90 μg of indicated cell lysate. At each indicated time point, the amount of reduced GSH was measured as described in the Methods Section. The results were normalized to a control without lysate. Data points marked with letters that are not the same are significantly different at p < 0.05.

We previously demonstrated that chiral α-naphthyl N-acetylalaninate probes cross the plasma membrane and were useful for detecting intracellular OPH activity [24]. We anticipated that S-NPAA would likewise cross the plasma membrane and cause GSH depletion. To test this hypothesis, we treated cultured cells with S-NPAA followed by GSH visualization with CMAC. CMAC reacts with intracellular GSH to produce a blue fluorescence. We also anticipated that GSH depletion would be most pronounced in cells with high expression of OPH activating enzyme. We found that S-NPAA crossed the plasma membrane and caused significant GSH depletion in LNCaP and COS-7-OPH cell lines and both these cell lines have high levels of OPH activity as semi-quantitatively indicated in Table 1 [24]. RWPE-1 and COS-7 cells have low OPH activity [24] and show low GSH depletion when treated with S-NPAA (Figure 5A). We analyzed the fluorescence levels between cell lines using ImageJ (Figure 5B) and found that GSH levels in S-NPAA treated LNCaP and COS-7-OPH cells were depleted at least two-fold compared to control cells with no S-NPAA: the COS-7-OPH cells and LNCaP cells showed about a three-fold and five-fold increase, respectively, in GSH depletion compared to that of RWPE-1 cells. The increased GSH consumption observed in the COS-7-OPH cells treated with S-NPAA compared to similarly treated COS-7 cells (Figure 5B) is particularly telling since the primary difference between these cells is overexpression of human OPH in the COS-7-OPH African green monkey kidney cells.

**Table 1:** **Summary of relevant data for cells treated with S-NPAA**

Cell line	OPH activity level*	GSH depletion with S-NPAA	Intrinsic oxidative stress**	Apoptosis with S-NPAA	Cell viability with S-NPAA
RWPE-1	+	+	+	+	+
LNCaP	++	+++	++	++	+++
DU145	+	+	++++	++	+++
PC3	+	+	+++++	++	+++
COS-7	+	+	NR	+	+
COS-7-OPH	+++++	+++++	NR	++	++

The number of + symbols indicates the fold increase of the observed condition compared to non-tumorigenic RWPE-1, e.g., COS-7-OPH cells have about five-fold more OPH activity than RWPE-1 cells. NR indicates that the condition has never been reported.

*From McGoldrick et al., [24].

**Intrinsic oxidative stress levels are summarized from Kumar et al., [3].

We next examined in vitro GSH depletion using lysates from the nontumorigenic RWPE-1 prostate epithelial cell line and the tumorigenic LNCaP, DU145 and PC3 prostate cell lines (Figure 5C). We found that LNCaP cells showed the highest depletion of intracellular GSH in all the prostate cells examined: a result consistent with our previously reported finding of high OPH activity/protein in this cell line as summarized in Table 1 [24].

### S-NPAA increases oxidative stress in cells with high OPH activity and promotes apoptosis in tumorigenic prostate cells

**Figure 6:**
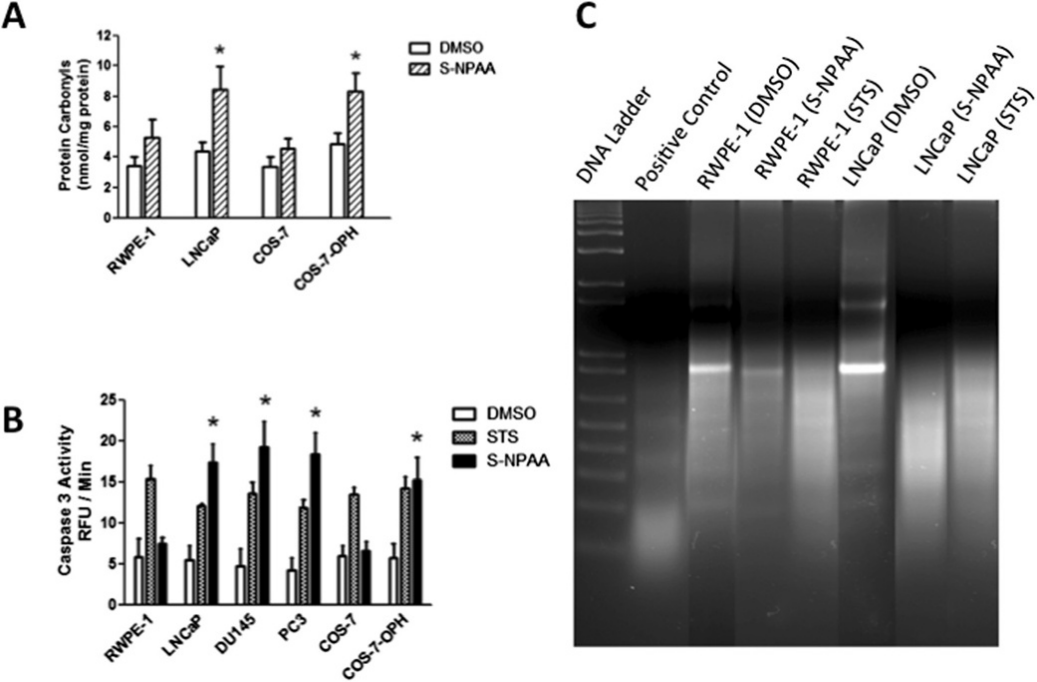
**LNCaP and COS-7-OPH show increased oxidative stress and apoptosis after treatment with S-NPAA. A)** RWPE-1, LNCaP, COS-7, and COS-7-OPH cell cultures were incubated with 25 μM S-NPAA for 6 hours. Protein carbonyl levels were measured in cellular lysates as described in the Methods Section. **B)** RWPE-1, LNCaP, DU145, PC3, COS-7, and COS-7-OPH cell cultures were incubated with 25 μM NPAA or 5 μM staurosporine (STS) as a positive control for 6 hours and caspase-3 activities in cellular lysates were measured as described in the Methods Section; *indicates that the S-NPAA treatment was significantly different from control (vehicle) at p < 0.05. **C)** RWPE-1 and LNCaP lysates were also examined for DNA fragmentation under the same conditions.

GSH is the primary intracellular antioxidant and plays a key role in maintaining cellular defense against oxidative stress, especially in cancer cells with high levels of intrinsic oxidative stress [10]. GSH depletion should, therefore, result in increased oxidative stress biomarkers in cells that are treated with S-NPAA. As shown in Figure 6A, we measured the level of protein carbonyls in RWPE-1, LNCaP, COS-7, and COS-7-OPH cells treated with S-NPAA for 6 hr. We found that S-NPAA-treated LNCaP and COS-7-OPH cells, with high OPH levels, had significantly higher protein carbonyl levels than similarly treated RWPE-1 and COS-7 cells with lower levels of OPH activity.

Kumar et al. [3] previously reported that tumorigenic LNCaP, DU145, and PC3 prostate cells had significantly higher intrinsic oxidative stress compared to non-tumorigenic RWPE-1 prostate cells. We therefore hypothesized that tumorigenic prostate cells, even those with low OPH activity, would undergo apoptosis after treatment with S-NPAA. We treated RWPE-1, LNCaP, DU145, PC3, COS-7, and COS-7-OPH cells with 25 μM S-NPAA for 6 hours and examined the caspase-3 activity levels of the cell lysates (Figure 6B). The cell lines were also treated with staurosporine, an ATPase inhibitor known to induce apoptosis and commonly used as a positive control in apoptosis studies. LNCaP, DU145, PC3, and COS-7-OPH cells had significantly more caspase-3 activity after treatment with S-NPAA compared to staurosporine-treated control cells. RWPE-1 and COS-7 cells showed no increase in caspase-3 activity after S-NPAA treatment. We then further confirmed the apoptosis-inducing ability of S-NPAA by examining DNA fragmentation, a hallmark feature of apoptosis, in treated RWPE-1 and LNCaP cells (Figure 6C). After treatment with S-NPAA, LNCaP cell lysates showed a high degree of DNA fragmentation while RWPE-1 cell lysates showed little DNA fragmentation. Increased caspase-3 activity and DNA fragmentation are consistent with cells undergoing apoptosis [27].

### S-NPAA decreases the cell viability of cells with high OPH activity and is dose dependent

**Figure 7:**
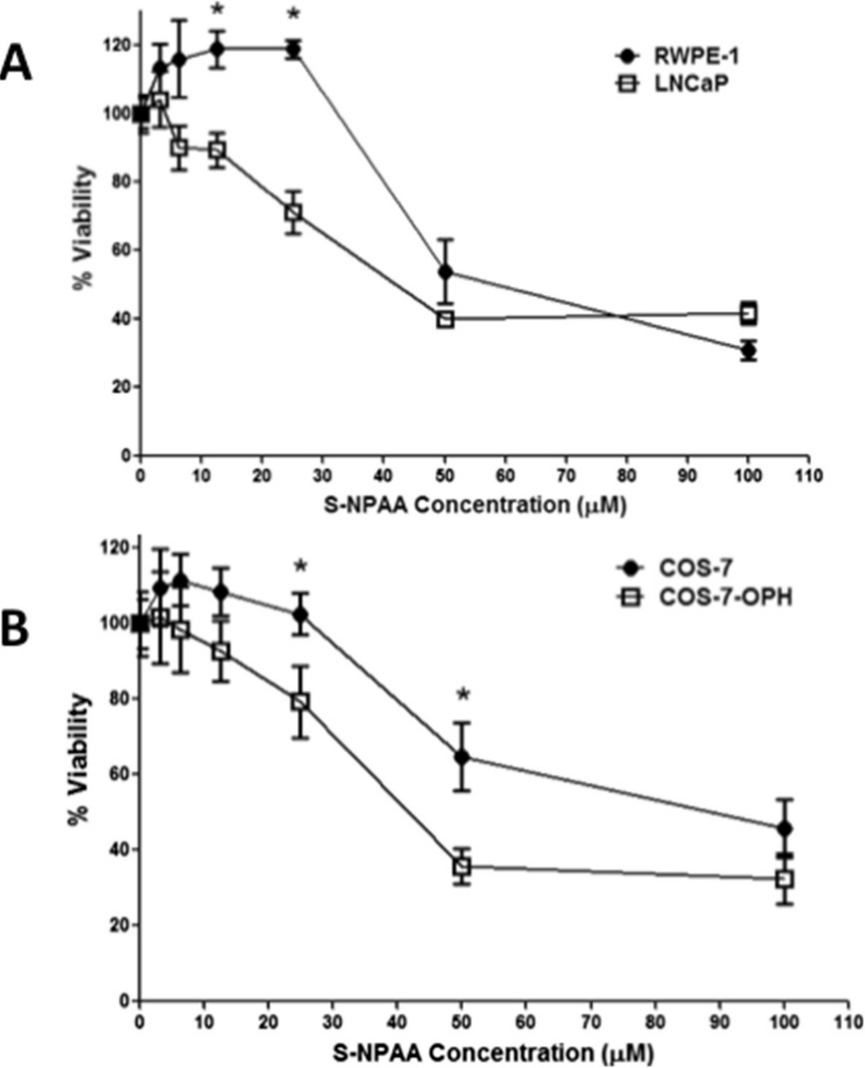
**LNCaP and COS-7-OPH cell viabilities were diminished more than RWPE-1 and COS-7 by S-NPAA. A)** RWPE-1 and LNCaP and **B)** COS-7 and COS-7-OPH cells were treated with the indicated doses of S-NPAA and incubated at 37°C for 24 hours. Cell viability was measured as a percent of control using a MTS viability assay; *indicates a significant difference between cell lines at p < 0.05.

We next examined the cell viability of RWPE-1, LNCaP (Figure 7A), COS-7, and COS-7-OPH (Figure 7B) cells after treatment with various single doses of S-NPAA. The MTS viability assay, a colorimetric method for determining the number of viable cells, was used to measure cell viability 24 hours after treatment. We found that single doses exceeding 30 μM NPAA were toxic to all four cell lines; however, the cell lines with high levels of OPH activity were more susceptible to S-NPAA at lower doses, i.e., 1.5 to 25 μM. At these lower doses, we found an approximately 10-30% decrease in LNCaP cell viability compared with RWPE-1 viability. Similar doses reduced viability of COS-7-OPH cells by 10-30% compared with COS-7 cell viability. In addition, we found that low doses of S-NPAA slightly increased cell proliferation in cells with low OPH activity.

Ideally, the prodrug should decrease the viability of tumorigenic cells such as LNCaP with little effect on non-tumorigenic cells such as RWPE-1 cells. Therefore, our follow-up experiments focused on trying multiple low doses of S-NPAA that might decrease cell viability in the tumorigenic prostate cell lines but cause minimal decrease in the viability of the nontumorigenic RWPE-1 cells.

### Multiple, low dose S-NPAA treatments decrease the cell viability of tumorigenic prostate cells with almost no effect on non-tumorigenic prostate cells

**Figure 8:**
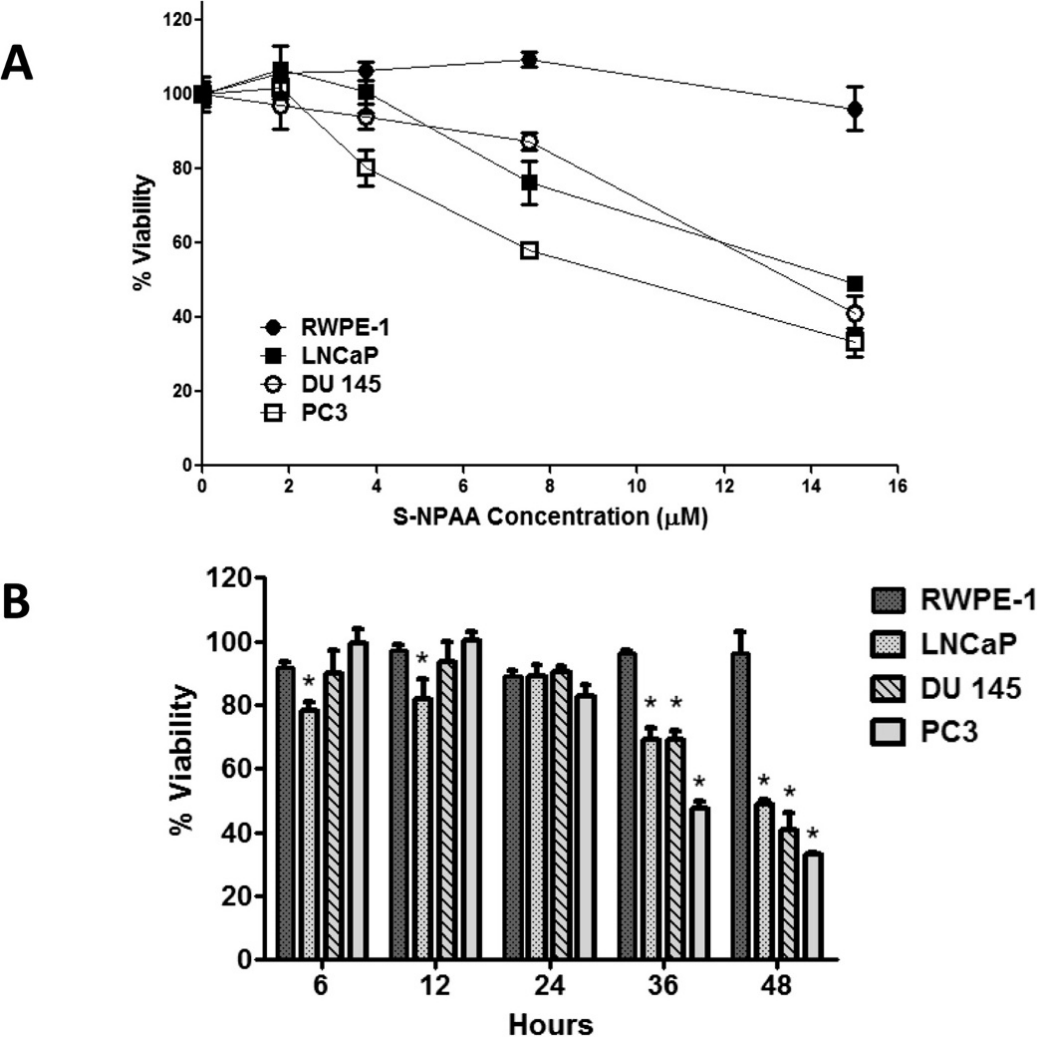
**Multiple low-dose treatments with S-NPAA significantly reduced tumorigenic cell viability. A)** Non-tumorigenic (RWPE-1) and tumorigenic prostate cells (LNCaP, DU145 and PC-3) were treated with the indicated doses of S-NPAA at 0 hours and given additional doses at 6, 12, 24, and 36 hours. Cell viability was measured as a percent of control at 48 hours using a MTS viability assay. Data points marked with letters that are not the same are significantly different at p < 0.05. **B)** Non-tumorigenic and tumorigenic prostate cells were treated with a 15 μM dose of S-NPAA at 0 hours and given additional 15 μM doses at 6, 12, 24, and 36 hours. Cell viability was measured at the indicated time point as a percent of control using a MTS viability assay; *indicates a significant difference between tumorigenic cells and RWPE-1 at p < 0.05.

We next examined a range of low dose concentrations of S-NPAA on tumorigenic and non-tumorigenic prostate cells administered at 0, 6, 12, 24 and 36 hours with cell viability measured after 48 hours. As shown in Figure 8A, the multiple low doses were quite effective at decreasing the viability of tumorigenic prostate cells but had almost no effect on the cell viability of non-tumorigenic RWPE-1 cells. Multiple doses of 7.5 μM S-NPAA reduced the viability of tumorigenic prostate cells (LNCap, DU145 and PC3) by 10-30% compared with RWPE-1. Multiple doses of 15 μM S-NPAA reduced the cell viability of tumorigenic prostate cells by 45-65% compared with RWPE-1 cells. We then examined the effects multiple doses of 15 μM S-NPAA at 0, 6, 12, 24 and 36 hours (see Figure 8B). We noted significant decreases in tumorigenic cell viability compared to RWPE-1 after 36 hours. After 48 hours, the viability of tumorigenic cells decreased to 45-65% compared with to the viability of untreated cells. At 6 and 12 hours, LNCaP cells began to show significant decreases (10-15% decrease) in viability compared to viability of untreated LNCaP cells. RWPE-1 viability levels were fairly constant with only about 5% variation among time points. These data suggest that repeated low doses of S-NPAA and the duration of treatment could be successfully modulated to preferentially inhibit the viability of tumorigenic prostate cancer cells with minimal effect on nontumorigenic prostate epithelial cells.

## Discussion

The work presented here suggests that the esterase activity of OPH can be exploited as a potential target for a novel chemotherapeutic QM generating N-acetyl-S-alaninate prodrug, S-NPAA (Figure 1). S-NPAA was found to deplete GSH in a manner completely analogous to that of NO-ASA, a well-characterized anti-cancer drug with minimal in vivo toxicity [18]. NO-ASA exerts an anticancer effect by depleting intracellular GSH and causing oxidative stress induced apoptosis by activation of the intrinsic death pathway [15]. As proof of concept, we found that OPH depletes GSH in the presence of S-NPAA in vitro as well as in cell lines overexpressing OPH (e.g. LNCaP or COS-7-OPH). Additionally, we found that S-NPAA, when activated by OPH, is effective at increasing oxidative stress (Figure 6A), inducing apoptosis (Figure 6B and C), and decreasing cell viability in tumorigenic prostate cancer cells while having only minimal such effects on a nontumorigenic prostate epithelial cell line (Figure 8A and B).

**Figure 9:**
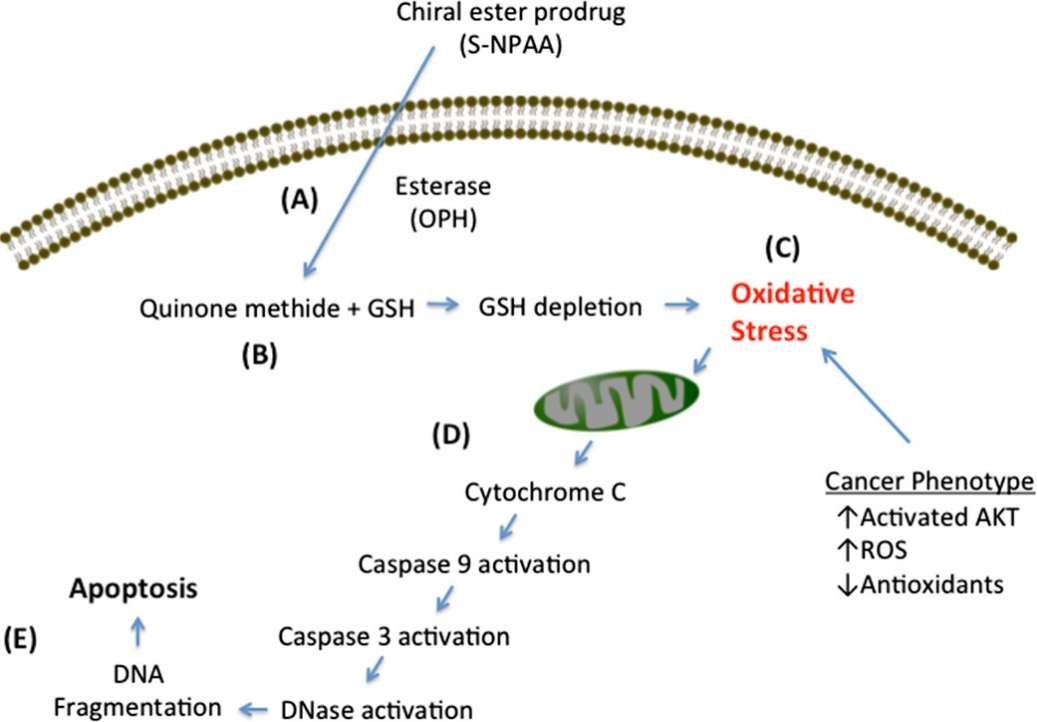
**Induction of cell death by a N-acetylalaninate QM producing prodrug. A)** The prodrug (S-NPAA) has been shown to cross the plasma membrane and be cleaved by the esterase activity of OPH. **B)** A quinone methide (QM) is generated which rapidly reacts with GSH causing a reduction in GSH levels. **C)** Cancer cells are known to typically have a pro-oxidant state due to activated Akt, which promotes the production of reactive oxygen species (ROS) and reduces some antioxidant defense mechanisms. The reduction in GSH levels by QM induces further oxidative stress that **D)** causes mitochondrial damage and activation of the caspase cascade, followed by **E)** DNA fragmentation and apoptosis.

As outlined in Figure 9, the work presented here suggests that S-NPAA can exploit a newly recognized weakness in one of the signaling pathways that cancer cells utilize to maintain an aggressive cancer phenotype, i.e., a high level of intrinsic oxidative stress due to the activation of the Akt kinase cascade. Akt kinase is a master component of the signaling cascades critical for regulating cell survival, cell growth, glucose metabolism, cell motility and angiogenesis [8]. Constitutive Akt activation is caused by mutations in components of its signaling cascade and results in cancer cells with an increased ability to escape apoptosis and proliferate. Moreover, Akt activation results in an increased production of cellular ROS which plays a causal role in maintaining many cancer phenotypes [28]. This, however, is a "double-edged sword" since the increased ROS production resulting from continuous Akt activation also results in an increased sensitivity to pro-oxidant drugs that can tip cancer cells into apoptosis [5].

We recently reported that OPH is overexpressed in the human LNCaP tumorigenic prostate cell line compared to control human (nontumorigenic) RWPE-1 prostate epithelial cells [24]. In the work reported here, we found that lysates from tumorigenic LNCaP cells were more effective at in vitro GSH depletion than non-tumorigenic RWPE-1 cell lysates (see Figure 5). The higher degree of GSH depletion in LNCaP cells is consistent with the overexpression of OPH found in these cells compared with that observed in the RWPE-1 cells (Table 1). LNCaP and COS-7-OPH cells treated with S-NPAA were also found to have a greater depletion of intracellular GSH (Figure 5A) as well as a greater loss of cell viability than similarly treated RWPE-1 and COS-7 cells (Figure 7). Collectively, our preclinical findings are significant since they potentially provide a molecular basis for potentially selecting those cancer patients most likely to respond to S-NPAA–like prodrugs, i.e., those with activated Akt and OPH overexpression.

While a high OPH activity contributes to the effectiveness of the S-NPAA prodrug, it is also plausible that a high level of basal cellular oxidative stress would similarly contribute to inhibiting cell growth even in the face of normal OPH activity. The apoptosis and viability studies reported here indicate that S-NPAA treatment is effective at decreasing viability not only in LNCaP cells, but also in the tumorigenic DU145 and PC3 human prostate cancer cells, which have OPH activities similar to RWPE-1 cells (Figure 8A and B). The high levels of intrinsic oxidative stress in PC3 and DU145 cells previously reported by Kumar et al. [3] might explain why S-NPAA is still effective in these cell lines. Table 1 provides a summary of our findings and those from Kumar et al. [3] and also shows the interrelationships between OPH activity and intrinsic oxidative stress, GSH depletion, apoptosis and cell viability after S-NPAA treatment. While high OPH activity appears to be sufficient to mediate apoptosis and loss of cell viability after treatment with S-NPAA, it is also clear that high levels of intrinsic oxidative in the tumorigenic prostate cell lines are also a key factor.

It is also likely that the basal antioxidant levels in cancer cells could be an additional variable that could influence the effectiveness of pro-oxidant drugs like S-NPAA. Cancer cell often have a high level of GSH to cope with their high level of intrinsic oxidative stress [29–31]. In addition to mutations resulting in Akt activation, mutations in antioxidant enzymes or mutations resulting in increased ROS production (e.g., many mitochondrial mutations) would also be important determinants of pro-oxidant drug efficacy.

The results of this study indicate that GSH-depleting QM-producing N-acetyl-L-alaninate ester prodrugs may be effective in the treatment of prostate cancer. The novel prototype ester prodrugs described here may also be effective as radiosensitizers for treating cancer tumors that are resistant to radiotherapy (e.g., melanomas), since cancer cells depleted of GSH are more sensitive to the cytotoxic effects of free radicals produced by ionizing radiation [29]. Moreover, isolated tumor stem cells that survive initial exposure to irradiation have been found to over-express genes controlling GSH biosynthesis but become sensitive to irradiation upon GSH depletion [30]. In addition, glutathione-S-transferases are often at elevated levels in tumor cells and these detoxifying enzymes can limit the effectiveness of some chemotherapeutic drugs by their covalent conjugation with GSH [31]. The GSH depleting prodrugs described in this study could potentially have a role in inhibiting the glutathione-S-transferase mediated elimination of some chemotherapeutic drugs.

## Conclusions

In conclusion, we have found that the four QM producing N-acetylalaninate ester prodrugs tested here are activated by OPH, and that S-NPAA is the most effective of the four prodrugs tested. Activation of S-NPAA by OPH leads to GSH depletion, increased oxidative stress, induction of apoptosis, and reduction of cell viability in tumorigenic prostate cells with little effect on non-tumorigenic RWPE-1 cells.
